# Stroke Patients Motivation Influence on the Effectiveness of Occupational Therapy

**DOI:** 10.1155/2018/9367942

**Published:** 2018-07-30

**Authors:** Jolita Rapolienė, Erika Endzelytė, Indrė Jasevičienė, Raimondas Savickas

**Affiliations:** ^1^Department of Rehabilitation, Medical Academy of Lithuanian University of Health Sciences, Kaunas, Lithuania; ^2^Department of Neurorehabilitation, Hospital of Lithuanian University of Health Sciences Kauno Klinikos, Kaunas, Lithuania

## Abstract

**Introduction:**

Individuals who have experienced stroke are often described as apathetic, having lost of interest, and unmotivated. This might be a problem in achieving treatment results. It is still unclear what impact age and gender have on the motivation.

**The Aim of Research:**

To evaluate motivation influence on the effectiveness of occupational therapy in patients with stroke.

**Methods:**

Study included 30 patients who experienced acute stroke. Multidimensional Health Locus of Control (MHLC) scale has been used for motivation assessment. Internal persons' motivation was evaluated to determine how much a person believes that his recovery depends on his own behaviour and external persons' motivation—how a person relates his state of health to the impact of the surrounding people. Performance of daily activities was assessed using Functional Independency Measure (FIM).

**Results:**

At the beginning of rehabilitation of the patients with stroke, external motivation was greater than the internal one. At the end of rehabilitation internal motivation has increased by 1.8±0.4 points, while the external motivation decreased by 2.4±0.6 points (p<0.05). At the beginning of rehabilitation patients independence in activities of daily living assessed with FIM was 70.0±2.9 points. At the end of rehabilitation their ability to perform daily activities improved by 26.9±1.9 points and reached 96.9±2.7 points (p<0.05). We found statistically significant (p<0.05) moderate correlation (r=0.72) between patients internal motivation at the beginning of the rehabilitation and increase of independence after rehabilitation.

**Conclusion:**

Older patients had lower internal motivation than younger ones, and independence in daily activities improvement was influenced by younger age and by brain damage depth.

## 1. Introduction

Individuals who have experienced stroke are often described as apathetic, having lost of interest, and unmotivated. This might be a problem in achieving treatment results. Factors associated with rehabilitation success include patients assertiveness, goal directedness, and presence of an intimate relationship. Motivation is the most important problem facing the rehabilitation professional. To foster motivation, therapists must be effective psychosocial advocates as well as functional coaches. Practitioners are just beginning to develop systematic methods to evaluate and enhance motivation in rehabilitation settings [1]. It is still unclear what impact age and gender have on motivation.* The aim of research *is to evaluate motivation influence on the effectiveness of occupational therapy in patients with stroke.

## 2. Literature Review

Mortality from stroke is decreasing; however, rate of patients experiencing long-term effects of stroke increases. Different sources indicate that stroke causes death up to 20 percent patients, more than 50 percent stroke survivors remain temporarily or permanently disabled, and about 10 percent affected individuals require long-term care. Only 20 percent of the working age individuals return back to work [2]. Cerebrovascular accident experienced patients are recovering long time; their condition is improving mainly during the first few months. Later, at 3-6 months status stabilizes, but recovery will continue for 1-2 years [2, 3]. Individuals who have experienced stroke are often described as apathetic, uninterested, and motiveless [3–5]. The term “motivation” in various fields of science is explained differently. In the modern psychology point of view, motivation is a whole of mental processes, encouraging the individual to act purposefully. It is a whole of factors which determine human behaviour. In physiology, motivation is described as conscious or unconscious needs, encouraging target related behaviour [3, 5, 6]. In rehabilitation motivation for activity is particularly important, since during rehabilitation patient's active participation is crucial. So it is very important that in the early stage of rehabilitation a person would be motivated to take part in rehabilitation. A number of studies analysing the factors that affect people who have suffered stroke have been made, rehabilitation efficiency. However, there is a lack of research, which explores the motivation influence on effectiveness of occupational therapy with stroke patients.

### 2.1. Statistical Analysis

Statistical analysis was performed using the software packages IBM SPSS 17. Quantitative variables were described using the mean, standard deviation (SD), and percentages (%). Comparison of two means was made using Student's* t*-test for dependent and independent samples. Associations between two variables were estimated using Pearson's correlation coefficient. Differences were accepted as statistically significant for values of p<0.05.

### 2.2. Methods

Study was approved by the clinic's Bioethics Center. All of the participants provided their written informed consent prior to their participation in the study, in accordance with the ethical principles of the Declaration of Helsinki. The study involved 30 people who experienced acute stroke (Table 1).

The study included patients who at the beginning of rehabilitation were evaluated using Mini-Mental State Examination (MMSE) and scored 21 points or more. To evaluate patients' motivation Multidimensional Health Locus of Control (MHLC) scale was used. All evaluations were made by researchers who applied occupational therapy. Occupational therapist evaluated each patient before treatment and after 20 occupational therapy procedures. Evaluated internal person's motivation determines how much a person believes that his recovery depends on his own behaviour and external person's motivation—how a person relates his state of health to the impact of the people surrounding him. Performance of daily activities was assessed using Functional Independency Measure (FIM). Subjects were evaluated at the beginning and at the end of rehabilitation. Special rehabilitation programs were created for patients based on their functional abilities and were administered by a team of rehabilitation specialists: physical medicine and rehabilitation physician, physical therapist, occupational therapist, speech therapist, social worker, psychologist, orthosis specialist, and nurses. The patient was encouraged to participate actively in the treatment program alongside with his family members. All subjects underwent individual occupational therapy 5 times a week. The length of single treatment was 30–40 minutes. Each patient in both groups received 20 occupational therapy procedures. The aims of occupational therapy were (1) hand movement training; (2) training of meaningful everyday activities; (3) promotion of motivation to perform activities. Hand movement training was carried out at the occupational therapy cabinet, using occupational therapy techniques and equipment. Bilateral arm movements training had been applied to patients with hemiplegia. Patients with hemiparesis performed tasks with affected hand. Patients were taught to perform self-care and mobility tasks, including correct hand movements while eating; specific movements performing dressing and personal hygiene. Developing mobility skills, balance was trained and patients were trained to perform safe transfers to bed, wheelchair, etc. Motivation to activities was promoted during the occupational therapy sessions by ensuring feedback.

During occupational therapy procedures, therapist encouraged patients to express and manage emotional upset related to illness and identified and addressed immediate concerns and patient's priorities and needs. Concern, responsiveness, and collaborative effort in the first few minutes set the tone and structure for all the treatment. Occupational therapist together with patient after stroke tried to formulate the main problem, its coping resources, and available solutions. The patient's hopes, ability to accept help and collaborate, and his own goal directed actions and self-evaluations were discussed, at the same time promoting realistic appraisal of rehabilitation possibilities. Occupational therapist emphasized the impact of personal effort on outcome in the treatment process and also invited the patient to express goals for treatment and for life after discharge. Concrete ADL objectives and broader life roles were discussed. Patients were encouraged to recognize that less is not necessary meaningless, defining the specific challenges of rehabilitation, promoting active coping and learning strategies, and at the same time promoting realistic progress appraisal. Positive feedback and reinforcement are essential therapeutic tools. Occupational therapist selected activities and interactions that helped patients in expressing and managing painful feelings; in sustaining performance of previously beneficial occupations and routines; in learning new skills for functional improvement, communication, stress management, and safety; in selecting activities that are remotivating by demonstrating the person's ability to accomplish goals and take care of himself or herself.

## 3. Results

At the beginning of rehabilitation, assessing the internal motivation, most of the patients agreed with the following statements: “if I get sick, it is my own behaviour which determines how soon I get well again” and “the main thing which affects my health is what I myself do”. At least patients agreed with the following statement: “When I get sick, I am to blame” (Table 2). At the end of rehabilitation, the average of internal motivation scores increased by 1.8±0.4 points (p<0.05). The most valued statement after rehabilitation remained the same, that is, “if I get sick, it is my own behaviour which determines how soon I get well again” which has statistically significantly (p <0.05) risen to 5.4±0.2 points. The worst (that means lowest scores in MHLC) subjects evaluated statement was “when I get sick, I am to blame” after rehabilitation which has statistically significantly (p<0.05) risen to 3.9±0.3 points (Table 2). Statistically significant difference between men and women internal motivation has not been obtained (p>0.5).

**Figure 1:**
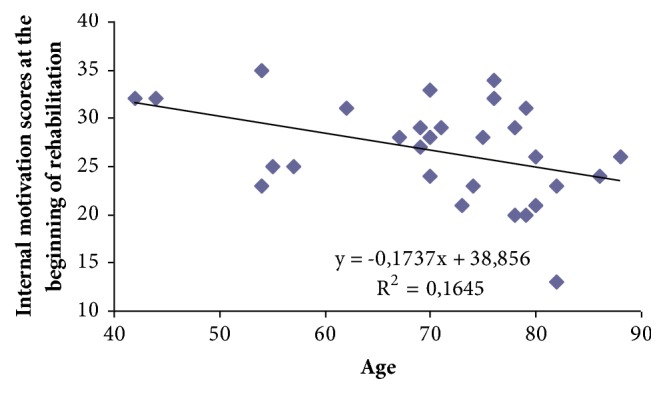
Correlation between age and internal motivation at the beginning of rehabilitation.

An analysis of the relationship between internal motivation and age showed statistically significant (p<0.05) and medium correlation (r=-0.41) between age and the size of internal motivation at the beginning of rehabilitation (Figure 1).

In elderly people, the internal motivation in the beginning of rehabilitation was lower. We found that the age of one-year internal motivation decreases by 0.2 points. At the beginning of rehabilitation, the average of subjects' external motivation was 30.2±0.8 points out of 36 possible points. Most of the subjects agreed with the following statement: “whenever I don't feel well, I should consult a medically trained professional”. At least people agreed with this statement: “my family has a lot to do with me becoming sick or staying healthy” (Table 2).

At the end of rehabilitation external motivation decreased (p<0.05). The statement “whenever I don't feel well, I should consult a medically trained professional” was valued at the beginning of rehabilitation (5.7±0.1). The approach to the statement “whenever I recover from an illness, it's usually because other people have been taking good care of me” has not changed significantly (p> 0.05). All other statements reflecting external motivation assessed during the rehabilitation statistically significantly decreased (p<0.05). Most has changed the attitude to the following statements: “health professionals control my health” (decreased by 0.7±0.2 points) and “having regular contact with my physician is the best way for me to avoid illness” (decreased by 0.6±0.2 points) (Table 2).

Prior to treatment patients' average FIM test results were 70.0 (±2.9). Most of the problems caused ability to perform daily activities: bathing, lower body dressing, and climbing the stairs. During these activities, individuals were required for maximal assistance. Moderate assistance was necessary for dressing the upper body, grooming, toileting, transferring at toilet and at shower or bath, and walking or using a wheelchair. Minimal assistance was sufficient while eating and transferring from bed, chair, or wheelchair. During the rehabilitation patients' ability to perform daily activities significantly improved by 26.9 ± 1.9 points (p <0.05). Activities, which at the beginning of rehabilitation required maximal assistance, at the end of rehabilitation were sufficient of minimal assistance. (Table 3).

There was no statistically reliable difference between men and women average FIM scores prior to treatment (p>0.05). Both men and women required maximal assistance for the same activities. At the end of the rehabilitation men's and women's independence in daily activities significantly (p<0.05) improved (Table 3). Statically significant difference between men's and women's independence scores was not found at the end of rehabilitation (p>0.05).

**Figure 2:**
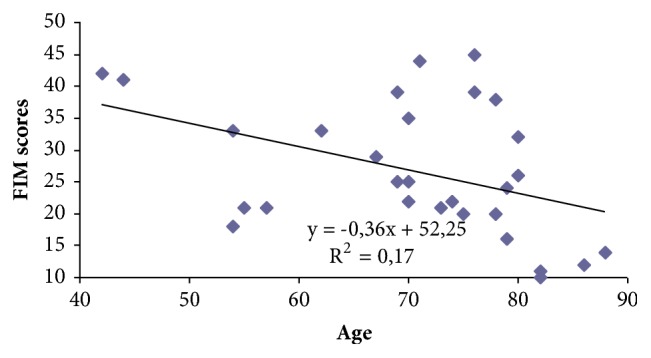
Correlation between patients' age and change of FIM scores during rehabilitation.

An analysis of the relationship between the change of patients' independence during the rehabilitation and patients' age showed a moderate negative (r=- 0.41) statistically significant (p<0.05) correlation (Figure 2).

Independence of older patients during rehabilitation increased less than the younger ones. We found that one year of age reduces the independence by 0.4 points. An analysis of data according to brain damage depth showed that, at the beginning of the rehabilitation individuals who had hemiplegia, FIM score average was 56.2 ± 1.5 points, while, for those who had hemiparesis, FIM score average was 64.5 ± 3.0. However statistically significant difference was not found (p>0.05). In both groups, activities of daily living at the end of rehabilitation statistically significant improved (p <0.05). For individuals who had hemiplegia, independence during rehabilitation course increased by 28.3 ± 3.8 points from 56.2 ± 1.5 to 84.5 ± 3.7 points, while the individuals who had hemiparesis – 26.5 ± 2.3 points from 64.5 ± 3.0 to 101.0 ± 2.7 points. A statistically significant difference (p<0.05) was obtained between independence scores at the end of the rehabilitation and brain damage depth.

**Figure 3:**
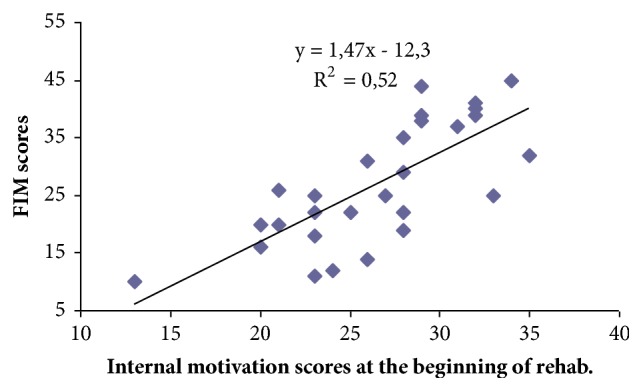
The relationship between internal motivation scores at the beginning of the rehabilitation and change of FIM scores in the course of rehabilitation.

After analysing the test data according to the affected side of the body, it was found that, for those who had damaged left side of the body, the average FIM score at the beginning of the rehabilitation was 73.9 ± 4.0 and those who had damaged right body side – 65.0 ± 4.1. But there was no statistically significant difference (p> 0.05). However, in the course of rehabilitation, a statistically significant improvement was observed in independence of daily activities (p <0.05). At the end of rehabilitation for patients who had damaged left side of the body FIM scores increased by 26.7 ± 2.6 points from 73.9 ± 4.0 to 100.6 ± 3.4 points, while for those who had damaged the right side of the body the FIM scores increased by 27.1 ± 2.8 points from 65.0 ± 4.1 to 92.1 ± 4.2 points. Still statistically significant difference between the affected side of the body and independence has not been determined (p<0.05). Assessing correlation between internal motivation scores at the beginning of rehabilitation and the change of independence during the course of rehabilitation, a significant and statistically important (p<0.05) moderate (r=0.72) correlation was observed. (Figure 3)

For people whose internal motivation points at the beginning of the rehabilitation were higher, performance of daily activities in the course of rehabilitation has improved more than those with lower internal motivation scores at the beginning of rehabilitation. We found that one score of the inner motivation at the beginning of rehabilitation improves patients' performance of daily activities by 1.5 points. The relationship between external motivation scores and change of FIM scores in the course of rehabilitation has not been established.

## 4. Discussion

Stroke causes various consequences, which disrupts the daily human life and functional independence and changes his life-sufficiency. After evaluation of subjects' motivation at the beginning of the rehabilitation we found that the average internal motivation score was 26.6 ± 0.9 points, while external motivation average was – 30.2 ± 0.8 points. They thought that their recovery is more dependent on doctors and other people than the impact of their own behaviour. At the end of rehabilitation internal motivation mean score statistically significant increased (p<0.05) by 1.8 ± 0.4 points, while external motivation mean score statistically significant decreased (p<0.05) by 2.4 ± 0.6 points. The survey data of MHLC questionnaire showed that patients believe that recovery depends on their own efforts, in the course of rehabilitation, increased, and the believe that physicians and others are responsible for their recovery decreased. According to the literature the increase in patients' age is associated with low motivation [3, 5, 7]. Our study also showed that older people were less motivated than younger ones. One year decreases internal motivation average score by 0.2 points. The literature indicates that individuals after stroke usually have difficulties in the following activities: the ability to independently use the toilet, bathing, climbing stairs, and the ability to walk or use a wheelchair and to dress lower part of the body [8]. After having evaluated the individuals' daily activities at the beginning of rehabilitation we found that most problems caused following activities: bathing (2.2±0.2), lower body dressing (2.3±0.2), and climbing the stairs (1.7±0.3). Performing these activities individuals required for maximal assistance. According to the literature findings, the course of rehabilitation most likely improves the ability to eat independently, perform grooming, dress the upper part of the body, move from bed to wheelchair and back, and, the latest, dress up the lower part of the body, bathe, and use the toilet independently [8]. After evaluating patients abilities to perform daily activities at the end of rehabilitation we found that activities that at the beginning of the rehabilitation required maximal assistance now required sufficient minimal assistance: bathing (4.1±0.2), lower body dressing (4.3±0.3), and climbing the stairs (3.8±0.2). Petruševičienė D. and A. Kriščiūnas assessed factors that influence the effectiveness of occupational therapy in patients with stroke and found that one of the factors was hemiplegia [7]. Our research showed that, for patients who had hemiplegia, performance of activities of daily living after rehabilitation increased by only 28.3±3.8 points from 56.2±1.5 to 84.5±3.7 points, while, for patients who had hemiparesis, it increased by 3.5±2.3 points from 64.5±3.0 to 101.0±2.7 points. In both groups, activities of daily living at the end of rehabilitation statistically significant improved (p<0.05). A statistically significant difference (p <0.05) was found between patients independence at the end of rehabilitation and nature of lesion. For individuals who had hemiplegia, FIM score average at the end of rehabilitation was lower than those who had hemiparesis. A. Juocevičius and colleagues assessed multiple rehabilitation effectiveness on elderly individuals with stroke and found that older people who come (p<0.05) and leave (p<0.01) rehabilitation are more functionally dependent [2]. The results of G. Bjørkløf and K. Engedal study showed that depressed older patients had a significantly higher external LOC orientation and used significantly less problem-focused coping strategies, compared with the nondepressed group [9]. Our study also showed a statistically significant (p<0.05) relationship between age and increased independence during the process of rehabilitation, since performing activities of daily living older people improved less than younger ones. One year of age FIM score change decreased by 0.4 points. In the literature motivation of patients with similar pathologies is often indicated as an important factor in the effectiveness of rehabilitation [3, 5, 10]. Motivated people better participate in the activities of rehabilitation and make greater progress than those who are less motivated [11]. Previous study conducted by Zulkifly and Ghazali stated that internal RLOC (IRLOC) and external RLOC (ERLOC) are significant predictors of physicals functioning. As a result, higher scores in IRLOC are correlated with patients having better physical functioning due to an individual self-efficacy that can lead them to recover more quickly from disability [12]. R. Cibulskienė investigated the motivation influence on occupational therapy and identified an obvious impact of motivation on increase in independence [13]. D. Jančiauskaitė investigated motivation influence on people's independence in occupational therapy and found that about 50 per cent. depends on the motivation [14]. Our study also showed an obvious influence on the motivation concerning improvement in patients' daily activities after stroke. There was a statistically significant (p<0.05) relationship between internal motivation scores at the beginning of the rehabilitation and FIM scores change in the course of rehabilitation. Even 52 percent of FIM score improvement depends on internal motivation at the beginning of rehabilitation. We found that one point of internal motivation at the beginning of rehabilitation improved performance of daily activities by 1.5 FIM points. The relationship between external motivation scores and FIM score change in the course of rehabilitation has not been determined. Therefore a survey revealed that people who believe that recovery depends on their own efforts and active participation in the rehabilitation process achieve much better results than those who are less convinced. Occupational therapist encouraging patient's motivation during the recovery process may increase the patients' confidence, which enhances their independence.

## 5. Conclusions

For individuals who have suffered stroke, external motivation was greater than the internal. In the course of rehabilitation, internal motivation increased and the external decreased. There was a statistically significant moderate correlation between individuals' internal motivation at the beginning of rehabilitation and improvement in activities of daily living during the rehabilitation process. Older patients' internal motivation was lower than younger ones, and the improvement of daily activities was influenced by younger age and lesion depth.

## Figures and Tables

**Table 1:** Demographics and characteristics of study subjects.

	n (%)
Men	12 (40%)
Women	18 (60%)
Age in years n (%)	
75-90	13 (43)
60-74	11 (37)
45-59	4 (13)
>44	2 (7)
Ischemic stroke	26 (87)
Haemorrhage into the brain	4 (13)
Hemiparesis	24 (80)
Hemiplegia	6 (20)

**Table 2:** The dynamics of internal and external motivation at the beginning and at the end of rehabilitation.

	BEGINING	END	*p*
	average	average	
INTERNAL MOTIVATION STATEMENTS			
1. If I get sick, it is my own behaviour which determines how soon I ge t well again.	5,2±0,2	5,4±0,2	p<0,05
6. I am in contro l of my health.	4,8±0,2	5,1±0,2	p<0,05
8. When I get sick, I am to blame.	3,3±0,4	3,9±0,3	p<0,05
12. The main thing which affects my health is what I myself do.	5,2±0,2	5,3±0,2	p>0,05
13. If I take care of myself, I can avoid illness.	4,0±0,2	4,4±0,2	p<0,05
17. If I take the right actions, I can stay healthy.	4,1±0,3	4,3±0,3	p>0,05
Overall:	26,6±0,9	28,4±0,8	p<0,05

Men's internal motivation	28.2±1.1	29.5±1.3	p<0,05
Women's internal motivation	25.6±1.3	27.7±1.0	p<0,05

EXTERNAL MOTIVATION STATEMENTS			
3. Having regular contact with my physician is the best way for me to avoid illness.	4.9±0.2	4.3±0.3	p<0.05
5. Whenever I don't feel well, I should consult a medically trained professional.	5.7±0.1	5.7±0.1	p<0.05
7. My family has a lot to do with me becoming sick or staying healthy.	4.2±0.3	3.7±0.3	p<0.05
10. Health professionals control my health.	4.8±0.2	4.1±0.2	p>0.05
14. Whenever I recover from an illness, it's usually because other people (for example, doctors, nurses, family, friends) have been taking good care of me.	5.5±0.1	5.4±0.1	p<0.05
18. Regarding my health, I can only do what my doctor tells me to do.	5.1±0.2	4.6±0.2	p>0.05
Overall:	30.2±0.8	27.8±0.7	p<0.05

Men's external motivation	31.6±1.0	28.5±0.8	p<0,05
Women's external motivation	29.3±1.1	27.3±.8	p<0,05

**Table 3:** Average FIM scores at the beginning and at the end of rehabilitation.

**ACTIVITIES**	**At the beginning**	**At the end**	*P value*
	average	average	
Eating	4.3±0.2	6.1±0.2	p<0.05
Grooming	3.2±0.2	5.5±0.2	p<0.05
Bathing/showering	2.2±0.2	4.1±0.2	p<0.05
Dressing upper body	3.0±0.2	5.4±0.2	p<0.05
Dressing lower body	2.3±0.2	4.3±0.3	p<0.05
Toileting	3.1±0.3	5.1±0.2	p<0.05
Bladder management	5.4±0.3	6.1±0.2	p<0.05
Bowel management	5.7±0.3	6.3±0.2	p<0.05
Transfers: bed/chair/wheelchair	3.9±0.3	5.6±0.3	p<0.05
Transfers: toilet	3.3±0.3	5.0±0.3	p<0.05
Transfers: bathtub/shower	3.0±0.3	4.4±0.3	p<0.05
Locomotion: walking/wheelchair	2.8±0.3	4.8±0.3	p<0.05
Locomotion: stairs	1.7±0.3	3.8±0.2	p<0.05
Comprehension	5.1±0.1	6.1±0.1	p<0.05
Expression	5.6±0.2	6.3±0.2	p<0.05
Social interaction	5.3±0.1	6.1±0.1	p<0.05
Problem solving	5.0±0.1	5.9±0.1	p<0.05
Memory	5.0±0.2	6.0±0.1	p<0.05
**Overall:**	70.0±2.9	96.9±2.7	p<0.05
Men's overall FIM scores	66.0±5.1	91.7±4.2	p<0.05
Women's overall FIM scores	22.7±3.5	100.4±3.4	p<0.05

## Data Availability

Any data presented in this article is available upon request from the corresponding author.
